# Serum GP73 is complementary to AFP and GGT-II for the diagnosis of hepatocellular carcinoma

**DOI:** 10.3892/ol.2013.1522

**Published:** 2013-08-09

**Authors:** SI-CONG HOU, MING-BING XIAO, RUN-ZHOU NI, WEN-KAI NI, FENG JIANG, XIAO-YAN LI, CUI-HUA LU, BU-YOU CHEN

**Affiliations:** 1 Department of Gastroenterology, Affiliated Hospital of Nantong University, Nantong, Jiangsu 226001, P.R. China; 2 Northern Jiangsu People’s Hospital, Yangzhou, Jiangsu 225001, P.R. China; 3 Department of Radiochemotherapy, Affiliated Hospital of Nantong University, Nantong, Jiangsu 226001, P.R. China

**Keywords:** time-resolved fluorescence immunological assay, golgi protein 73, hepatocellular carcinoma

## Abstract

Golgi protein 73 (GP73) is a resident Golgi type II transmembrane protein that has been reported to markedly increase in chronic liver disease, particularly in hepatocellular carcinoma (HCC). However, it remains unclear as to whether serum GP73 represents a reliable serum marker for the diagnosis of HCC. The aim of the present study was to evaluate the diagnostic value of serum GP73 in patients with HCC and to determine the diagnostic accuracy of measuring serum GP73 in combination with α-fetoprotein (AFP) and γ-glutamyl transferase isoenzyme II (GGT-II) in HCC. Serum GP73 was detected using a time-resolved fluorescence immunological assay (TRFIA) and enzyme-linked immunosorbent assay (ELISA) in 79 HCC cases, including 16 liver cirrhosis, 30 chronic hepatitis and 28 healthy individuals. The correlation between serum GP73 and tumor size and HCC grading was analyzed and the complementary diagnostic value of serum GP73, AFP and GGT-II was evaluated. TRFIA was established for the detection of serum GP73 and was sensitive and reproducible. The expression levels of serum GP73 were markedly higher in the patients with HCC when compared with those of the individuals with liver cirrhosis and chronic hepatitis or the healthy individuals. According to the receiver operating characteristic (ROC) curve, diagnostic sensitivity and specificity for HCC with a cut-off value of 78.1 ng/l were 73.4 and 79.0%, respectively. However, no correlation was identified among serum GP73 and tumor size or grading, and no correlations were identified among serum GP73, AFP and GGT-II. The diagnostic sensitivities for HCC, as detected by TRFIA of GP73, AFP and GGT-II, were 73.4, 55.6 and 68.4%, respectively, and the specificities were 80.0, 86.7 and 97.1%, respectively. The combined determination of these markers increased the diagnostic sensitivity to 96.3% for HCC. TRFIA functions as a sensitive and replicable assay for the detection of serum GP73. The levels of serum GP73 were significantly higher in the HCC group when compared with the individuals with benign liver diseases. Serum GP73 may serve as a potential independent diagnostic candidate for HCC and the combined determination of serum GP73, AFP and GGT-II may increase the diagnostic efficiency of HCC.

## Introduction

Hepatocellular carcinoma (HCC) is the most common type of primary liver cancer and represents the third leading cause of cancer-related mortality worldwide. HCC is particularly prevalent in Asia and Africa (1,2). The majority of HCC cases are detected at advanced stages of the disease, which precludes the use of curative surgical therapy. The prognosis of HCC is generally poor and the mortality rate is similar to the incidence rate. Consequently, detection of early-stage HCC remains an effective approach to substantially improve the overall outcome of individuals with HCC (3). The diagnosis of HCC is usually determined via tumor biomarkers in serum and by instrumental tests, including hepatic ultrasonography, computed tomography, magnetic resonance imaging and biopsy. Although a number of studies have investigated positive cancer biomarkers for HCC, none have been identified as the optimal choice. At present, α-fetoprotein (AFP) detection has been widely adopted for the diagnosis of HCC despite its low sensitivity (4–6). The use of AFP in combination with additional serum markers, including lens culinaris agglutinin reactive AFP (7), golgi protein 73 (GP73) (8) and γ-glutamyl transferase (GGT-II) (9), is likely to significantly increase the sensitivity compared with AFP alone.

GP73 is a resident golgi type II transmembrane protein expressed primarily in human epithelial cells (10). In the normal human liver, GP73 is expressed in biliary epithelial cells, but detection is negligible in hepatocytes. However, upregulated expression of GP73 has been identified in hepatic cells in liver disease (11). Previous studies have also shown increased serum GP73 levels in patients with chronic liver disease and, in particular, in HCC patients. This phenomenon may be due to migration of the GP73 protein to the plasma membrane and diffusion into the circulation (12,13). Thus, GP73 has been hypothesized to represent a novel serum marker for HCC. In addition, previous studies have proposed that GP73 exhibits a diagnostic ability superior to that of AFP (14,15). Therefore, it is reasonable to hypothesize that the determination of GP73, combined with additional significant HCC serum markers, may enhance diagnostic accuracy.

The aim of the present study was to compare the expression levels of serum GP73 in control and patient groups of individuals with liver diseases, including chronic hepatitis, liver cirrhosis and HCC, to evaluate and investigate the diagnostic value and accuracy of measuring serum GP73 in combination with AFP and GGT-II in HCC patients.

## Patients and methods

### Patient selection

A total of 184 serum samples were obtained from the Affiliated Hospital of Nantong University (Nantong, China), including 79 HCC (median age, 53 years old; 51 males and 28 females), 47 liver cirrhosis (median age, 43 years old; 27 males and 20 females), 30 chronic hepatitis (median age, 57 years old; 23 males and 7 females) and 28 healthy (median age, 50 years old; 14 males and 14 females) individuals. The blood samples of the HCC patients were collected prior to any interventions, including surgery or radiochemotherapy. A portal vein thrombosis (PVT) was identified in 27/79 HCC cases and the tumor diameters were <5 and ≥5 cm in 23 and 56 cases, respectively. This study was approved by the ethics committee of the Affiliated Hospital of Nantong University (Nantong, China). Written informed consent was obtained from the patients.

### Time-resolved fluorescence immunological assay (TRFIA) of serum GP73

Optimal concentrations of the reagents for TRFIA were determined according to experimental data in a trial-and-error procedure. Each well was coated with 100 μl mouse monoclonal antibodies against GP73 (4 mg/l; Santa Cruz Biotechnology, Inc., Santa Cruz, CA, USA) prior to overnight storage at 4°C. The plates were washed 4 times with 300 μl/well PBS and 0.05% Tween 20, followed by incubation with 250 μl/well blocking solution (PBS containing 10% FCS) at 4°C for 48 h. The blocking solution was poured off and 50 μl serum samples, negative controls and a series of GP73 standard dilutions were loaded into the appropriate wells and incubated at 37°C for 4 h. Subsequently, the plates were washed four times with PBS, and 100 μl goat anti-human polyclonal antibodies conjugated with biotin (80 μg/l; Santa Cruz Biotechnology, Inc.) were added to each well. Plates were agitated on an orbital shaker (Stuart Equipment, Stone, UK) at 1.5 × g, incubated at room temperature for 2 h and washed 4 times prior to adding 100 μl europium-labeled streptavidin (500 μg/l; Perkin-Elmer, Waltham, MA, USA) to each well. Following 1-h incubation at room temperature, the plates were washed 4 times as described and 200 μl enhancement solution was added to each well prior to a 15-min rotating incubation at room temperature in the dark. The plates were read using a Victor™ X5 automatic time-resolved fluorescence detector (Perkin-Elmer).

### Enzyme-linked immunosorbent assay (ELISA) detection of serum GP73

Serum GP73 was measured using ELISA kits obtained from Yifeng Biotechnology Co., Ltd., (Shanghai, China) according to the manufacturer’s instructions. Briefly, 50 μl samples were loaded onto ELISA plates precoated with monoclonal anti-GP73 and incubated at 37°C for 45 min. Subsequent to being washed four times with washing buffer, 50 μl/well anti-IgG conjugated with biotin was added and then the samples were incubated at 37°C for 30 min. The plates were washed again, streptavidin-HRP solution was added and the samples were incubated at 37°C for 30 min. Color developing agents A and B (50 μl/well) were added in sequence and incubated in the dark for 15 min. The reaction was terminated with stop buffer. Absorbance was measured at 450 nm on a microplate reader. The concentrations of GP73 were determined by interpolation from the standard curve.

### Determination of serum AFP and GGT-II

The presence of AFP was determined by chemiluminescence immunoassay and the reagents were obtained from Abbott Laboratories (Chicago, IL, USA). Serum GGT-II was determined using polyacrylamide gel electrophoresis kits (Jiangsu Zongheng Co., Ltd., Nantong, China), as described previously (16).

### Statistical analysis

Stata View 7.0 software package (Stata Corp LP, College Station, TX, USA) was used for the statistical analysis. Serum GP73 levels are presented as the median (range) and were analyzed with a Wilcoxon rank sum test. χ^2^ or Fisher’s exact tests were performed for any 2×2 tables. The correlation between serum GP73 and AFP and GGT-II was determined by Spearman’s rank correlation. Receiver operating characteristic (ROC) curves were used to evaluate the diagnostic value of the serum markers. P<0.05 was considered to indicate a statistically significant difference.

## Results

### Distribution of serum GP73 determined by TRFIA or ELISA

**Figure 1:**
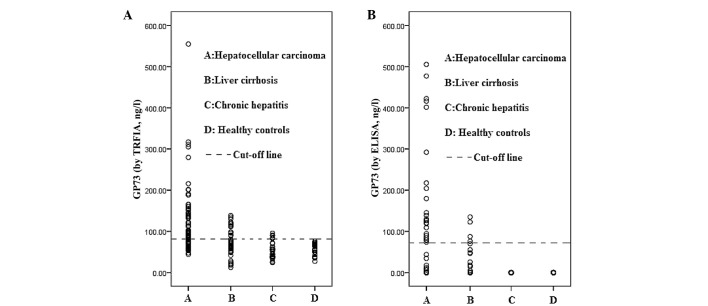
Scatterplot of serum GP73 levels, as detected by (A) TRFIA and (B) ELISA. GP73, golgi protein 73; TRFIA, time-resolved fluorescence immunological assay; ELISA, enzyme-linked immunosorbent assay.

Each blood sample was assessed for the GP73 levels using TRFIA and ELISA. The median (range) values of the GP73 levels in the HCC, liver cirrhosis, chronic hepatitis and healthy controls were 95.5 (43.9–554.8 ng/l), 69.3 (12.4–138.5 ng/l), 63.2 (27.2–95.6 ng/l) and 50.4 (24.1–75.8 ng/l), respectively, as determined by TRFIA. The serum GP73 levels in the HCC samples were markedly higher when compared with the benign liver disease and healthy control samples. The median levels of GP73, as determined by ELISA, were 0 ng/l for all groups, indicating that the sensitivity of ELISA for serum GP73 was considerably lower when compared with that of TRFIA. However, ELISA also identified higher levels of GP73 in the HCC patients compared with the additional groups (Table I; Fig. 1).

**Figure 2:**
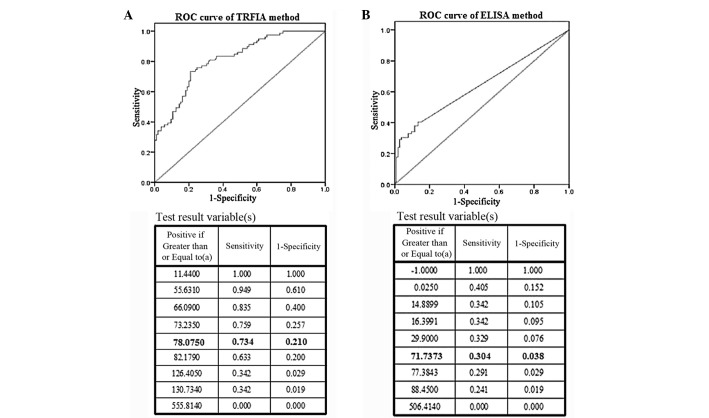
ROC curves of serum GP73 as detected by (A) TRFIA and (B) ELISA for the diagnosis of HCC. HCC, hepatocellular carcinoma; ROC, receiver operating characteristic; TFRIA, time-resolved fluorescence immunological assay; ELISA, enzyme-linked immunosorbent assay; HCC, hepatocellular carcinoma.

In addition, ROC curves were plotted to determine the optimal cut-off values to identify the sensitivity and specificity of serum GP73 in the patients with HCC. Accuracy was measured by the area under the ROC (AUROC) curve. The AUROC curve by TRFIA for GP73 was 0.814 (95% CI, 0.753–0.874) and the sensitivity and specificity, with cut-off values of 78.1 ng/l, were 73.4 and 79.0%, respectively (Fig. 2). The AUROC curve by ELISA for GP73 was 0.643 (95% CI, 0.559–0.725) and the sensitivity and specificity, with cut-off values of 71.7 ng/l, were 30.4 and 96.2%, respectively (Table II; Fig. 2).

The correlation between the serum GP73 levels and a number of tumor grading parameters was also evaluated, and the statistical analyses identified no correlations between the serum GP73 levels and the tumor size or PVT tumors (P>0.05; Table III).

### Serum AFP and GGT-II in HCC

**Figure 3:**
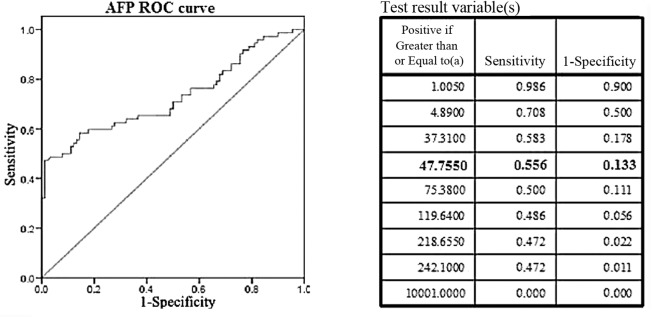
ROC curve of AFP serum levels for the diagnosis of HCC. ROC, receiver operating characteristic; AFP, α-fetoprotein; HCC, hepatocellular carcinoma.

The serum AFP level was significantly higher in the HCC group compared with the additional groups (P<0.05). The median serum AFP level in HCC was 270.00 ng/ml, which was higher compared with the liver cirrhosis (7.26 ng/ml), chronic hepatitis (13.80 ng/ml) and healthy control (2.33 ng/ml; Table IV) samples. According to the ROC curves, the sensitivity and specificity of GP73, with cut-off values of 47.8 ng/ml, were 55.6 and 86.7%, respectively (Table IV; Fig. 3). The sensitivity of the serum GGT-II levels for the diagnosis of HCC was also evaluated and was 68.4% in the HCC patients (Table IV).

### Serum GP73 levels are complementary to the serum levels of AFP and GGT-II

Spearman’s rank correlation test was performed on the serum GP73, AFP and GGT-II levels. No correlations were identified between the serum GP73 levels and AFP (r=0.0920; P=0.5384) or GGT-II (r=0.1321; P=0.3763), indicating that the three serum markers may have complementary roles in the diagnosis of HCC. The sensitivities of GP73, AFP and GGT-II for the diagnosis of HCC were 73.4, 55.6 and 68.4%, respectively, however, the combination of the three markers may increase the sensitivity to 96.3% (Table V).

## Discussion

The present study demonstrated that patients with HCC exhibit markedly higher levels of GP73 in the serum compared with patients with chronic hepatitis, liver cirrhosis and healthy controls. No correlations were identified among serum GP73 levels and the additional parameters, including tumor size and grading. In addition, no correlations were identified between the serum levels of AFP, GGT-II and GP73. The detection of serum GP73 in combination with classic HCC tumor markers, AFP and GGT-II, may improve the diagnostic power for HCC. We hypothesized that GP73 may represent a serum marker for HCC.

The early diagnosis and treatment of HCC is vital for improving the overall survival rate of patients (17). Routine surveillance is generally recommended worldwide for patients at risk of developing HCC, including individuals with chronic hepatitis and liver cirrhosis. At present, serum AFP and abdominal ultrasonography are the most common tools adopted for the diagnosis and monitoring of HCC in clinical practice. However, the clinical value of AFP, also referred to as the ‘gold-standard’ biomarker of HCC, remains controversial, as elevated AFP levels are often detected in patients with benign liver disease and additional malignancies, but not in a large proportion of early-stage HCCs (18). The clinical value of AFP has previously been challenged due to its low sensitivity and specificity (4–6).

A hepatoma-specific band of serum GGT-II has been determined to be an effective tumor marker complementary to AFP for the diagnosis of HCC. In 1985, following 10 years of follow-up, Xu *et al* reported GGT-II-positive expression in 90% (81/90) of HCC cases and no expression in the majority of patients with acute and chronic viral hepatitis, extrahepatic tumors, pregnant women and healthy controls (19). In addition, GGT-II-positive expression was identified in 74.0% (89/120) of HCC (sensitivity), 17.78% (16/90) of cirrhosis and 43.8% (14/32) of small HCC cases, indicating that GCT-II may represent an effective tumor marker for the diagnosis of HCC (9).

Consistent with the present results, no correlation has been reported between the levels of serum expression and AFP and GGT-II in previous studies (20). It has been indicated that the simultaneous determination of AFP and GGT-II may improve diagnostic accuracy for HCC (9). Previously, additional serum biomarkers, including DCP, AFU and GPC3, have been widely investigated, however, none have been identified as optimal for the early detection of HCC. A number of clinical and experimental studies have shown the significance of serum GP73 expression in liver diseases, particularly in HCC. In 2005, Block *et al* reported that increased serum GP73 levels were detected in patients with hepatitis B virus-related HCC (12). Similarly, Marrero *et al* demonstrated that serum GP73 levels were significantly increased in patients with hepatitis C virus-related HCC compared with cirrhotic controls (15). In addition, serum GP73 levels have been shown to be more sensitive at the early stages of HCC (14), and Liu *et al* reported that GP73 may represent an effective serum marker for monitoring the progression of liver diseases (21). In a previous Chinese study, a significant correlation was identified between the overexpression of GP73 at the protein and/or mRNA levels and the aggressive behavior of HCC. However, no correlation was reported with the overall patient survival rates (22). In addition, Mao *et al* identified that GP73 may also be utilized for the surveillance of HCC recurrence in post-operative patients (17). To investigate the diagnostic abilities of serum GP73 and AFP for HCC, Zhou *et al* performed a diagnostic meta-analysis with the following results: Sensitivity, 76 (95% CI, 51–91%) vs. 70% (95% CI, 47–86%) and specificity, 86 (95% CI, 65–95%) vs. 89% (95% CI, 69–96%), respectively, This indicated that serum GP73 has a comparable accuracy to AFP for the diagnosis of HCC (8). The elevated levels of GP73 expression identified in HCC may be due to an increase in the cytokine response and the level of the viral infection itself. Kladney *et al* identified that GP73 expression increased in response to interferon γ and that it was inhibited by tumor necrosis factor α (11). Kawamoto *et al* demonstrated that α1,6-fucosyltransferase enhanced the expression of GP73 (23). However, the precise mechanism of GP73 elevation in HCC remains unclear.

TRIFA was used in the current study to determine the level of serum GP73 and was validated as an ideal measure for serum GP73 in liver diseases, particularly in HCC, due to its high sensitivity. The diagnostic agreement between ELISA and TRFIA was evaluated using various cut-off values, according to ROC curves. Although ELISA kit detection is extensively used, the sensitivity of serum GP73 detection, with a cut-off value of 78.1 ng/l, by TRIFA was notably higher when compared with that of ELISA in HCC. The higher sensitivity of TRFIA was likely to be due to the use of a highly detectable ‘tracer’ The sensitivity of labeled reagent techniques may be substantially increased by improving the signal-to-noise ratio. In comparison to conventional methods, TRFIA technology is simple to perform, reproducible, accurate and amenable to automation. Therefore, due to its improved analytical ability, we hypothesized that TRIFA represents an alternative to traditional technologies for the sensitive determination of serum GP73 in HCC.

In the present study, the sensitivities of GP73, AFP and GGT-II were 73.4, 55.6 and 68.4%, respectively. The differences in the sensitivity of these markers was likely to be due to the various synthetic and secretion pathways in the cells. Of the 3 markers, GP73 exhibited the highest sensitivity (73.4%) and GGT-II the highest specificity (97.1%). The diagnostic accuracy of GP73 in combination with the conventional serum markers, AFP and GGT-II, was improved and has been demonstrated by ROC analysis. Previously, no studies have analyzed the correlation between the serum expression levels and GGT-II and GP73 in HCC patients, and no correlation was identified in the present novel study. However, the results indicated that the combined determination of the three complementary markers represents a higher sensitivity level for the diagnosis of HCC, but the discriminating ability of these markers must be verified in an additional cohort of HCC patients by investigating sensitivity and specificity independently. The bootstrap estimate of bias in the AUROC was relatively small and therefore, the resulting AUROC showed only a slight decrease. In conclusion, the current data obtained, with regard to sensitivity and specificity, may serve as a measurement of the diagnostic ability.

## Figures and Tables

**Table I:** Serum GP73 levels, as detected by TRFIA and ELISA.

		TRFIA (ng/l)			ELISA (ng/l)		
			
Subgroups	n	Median (range)	Z	P-value	Median (range)	Z	P-value
HCC	79	95.5 (43.9–554.8)			0 (0–505.4)		
Liver cirrhosis	47	69.3 (12.4–138.5)	4.152	0.000	0 (0–134.9)	1.978	0.045
Chronic hepatitis	30	63.2 (27.2–95.6)	6.085	0.000	0 (0–0.1)	3.578	0.000
Healthy controls	28	50.4 (24.1–75.8)	6.255	0.000	0 (0–0.0)	3.921	0.000

Z and P-values were calculated by a Wilcoxon rank sum test. P<0.05, vs. HCC group. GP73, golgi protein 73; TFRIA, time-resolved fluorescence immunological assay; ELISA, enzyme-linked immunosorbent assay; HCC, hepatocellular carcinoma.

**Table II:** Diagnostic sensitivities of GP73 (ng/l), as detected by TRFIA and ELISA.

Subgroups	Total, n	TRFIA^b^		ELISA^c^	
	
		n	%	n	%
HCC	79	58	73.4^a^	25	31.6^a^
Liver cirrhosis	47	16	34.0	4	8.5
Chronic hepatitis	30	5	16.7	0	0.0
Healthy controls	28	0	0.0	0	0.0

a P<0.05, vs. all subgroups, respectively;

b ≥71.7 and

c ≥78.1 cut-off values.

χ^2^ test or Fisher’s exact test. GP73, golgi protein 73; TFRIA, time-resolved fluorescence immunological assay; ELISA, enzyme-linked immunosorbent assay; HCC, hepatocellular carcinoma.

**Table III:** Correlation between GP73 levels and tumor size and PVT in 79 HCC patients, as detected by TRFIA.

Groups	n	GP73 positive, n	GP73 positive rate, %
Tumor size, cm			
<5	23	14	60.9^a^
≥5	56	44	78.6
PVT			
+	27	23	85.2^b^
−	52	35	67.3

Statistical analyses were performed by χ^2^ test.

a P>0.05, vs. ≥5cm group;

b P>0.05, vs. PVT(−) group.

PVT, portal vein tumor thrombus; GP73, golgi protein 73; TFRIA, time-resolved fluorescence immunological assay; HCC, hepatocellular carcinoma.

**Table IV:** Serum AFP and GGT-II levels in various hepatic diseases and healthy controls.

	AFP (ng/ml), CLEIA					
		
Subgroups	n	Median (range)	Z	P-value	≥47.8, n (%)	GGT-II positive, n (%)
HCC	79	270.00 (0.65–10,001.00)			44 (55.6)^a^	54 (68.4)^a^
Liver cirrhosis	47	7.26 (0.38–860.68)	4.723	0.000	12 (25.5)	3 (6.4)
Chronic hepatitis	30	13.80 (0.28–237.31)	4.839	0.000	2 (6.7)	0 (0.0)
Healthy controls	28	2.33 (0.21–9.80)	5.898	0.000	0 (0.0)	0 (0.0)

Z and P-values were calculated by a Wilcoxon rank sum test, HCC group vs. the additional groups, respectively;

a P<0.05, vs. additional groups.

Statistical analyses were performed by χ^2^ test. AFP, α-fetoprotein; GGT-II, γ-glutamyl transferase isoenzyme II; CLEIA, chemiluminescence immunoassay; HCC, hepatocellular carcinoma.

**Table V:** Complementary values of GP73, AFP and GGT-II for HCC diagnosis.

	Sensitivity, %	Specificity, %	Accuracy, %
GP73 (TRFIA)-positive	73.4 (58/79)^a^	80.0 (84/105)	77.2 (142/184)
AFP-positive	55.6 (44/79)^a^	86.7 (91/105)	73.4 (135/184)
GGT-II-positive	68.4 (54/79)^a^	97.1 (102/105)	84.8 (156/184)
a+b	88.6 (70/79)	69.5 (73/105)	77.7 (143/184)
a+c	91.1 (72/79)	77.1 (81/105)	83.2 (153/184)
b+c	86.1 (68/79)	84.8 (89/105)	85.3 (157/184)
a+b+c	96.2 (76/79)	67.6 (71/105)	79.9 (147/184)

a, GP73 (TRFIA)-positive; b, AFP-positive; c, GGT-II-positive.

a P<0.05, a, b and c vs. a+b+c, respectively.

Statistical analyses were performed by χ^2^ or Fisher’s exact tests. GP73, golgi protein 73; AFP, α-fetoprotein; GGT-II, γ-glutamyl transferase isoenzyme II; TFRIA, time-resolved fluorescence immunological assay; HCC, hepatocellular carcinoma.
